# ‘Anything that We Can Do to Help, It’s Got to Be Good’ The Everyday Pragmatism of NHS Charities

**DOI:** 10.3167/saas.2026.340105

**Published:** 2026-03-01

**Authors:** Francesca Vaghi

**Keywords:** Angleterre, bénévolat, charité, crise, État-providence, système médical, charity, crisis, England, healthcare system, voluntarism, welfare state

## Abstract

This article explores charity and voluntarism in England’s NHS during a period of ‘extended crisis’. Based on ethnographic research with an NHS charity, it examines how charitable organisations fulfil a dual role, by supplementing healthcare services and providing a ‘safety net’ to volunteers. Through the concept of ‘everyday pragmatism’, the article explains paid staff and volunteers’ motivations to improve present circumstances, while imagining a better future. The discussion contributes to wider debates about the reconfiguration of welfare states, suggesting that charity and voluntarism within the NHS are imperfect solutions that nonetheless genuinely seek to redress inequalities. Ultimately, everyday pragmatism describes people’s motivations to act not only despite the circumstances, but in acknowledgement of them.

## Résumé

Cet article explore la charité et le bénévolat dans les services nationaux de santé en Angleterre dans une période de ‘crise étendue’. En s’appuyant sur la recherche ethnographique avec un service national de santé, nous examinons comment les organismes de charité remplissent un double rôle, en soutenant les services médicaux et en fournissant un ‘filet de sécurité’ aux bénévoles. Au travers le concept de ‘pragmatisme de tous les jours’, l’article explique les motivations des salariés et des bénévoles pour améliorer les circonstances actuelles et pour imaginer un meilleur avenir. La discussion contribue aux débats plus conséquents sur la reconfiguration des États-providence, en proposant que la charité et le bénévolat dans les services nationaux de santé sont des solutions imparfaites qui visent à réduire les inégalités. En fin de compte, le pragmatisme de tous les jours décrivent les motivations de gens de pouvoir agir non seulement à cause de circonstances mais aussi en reconnaissance de ces circonstances elles-mêmes.

Worldwide, crisis no longer seems to represent a momentary state of emergency, but rather an ongoing situation that is continually defining our present. In 2008, in a seminal article on the temporality of crisis, Henrik Vigh wrote that, ‘No longer temporary [crisis] becomes instead a state of ordered disorder … Instead of being a passing period of chaos it settles as a social state’ (2008: 12–13). Lauren Berlant echoed this perspective in *Cruel Optimism*, stating that ‘the present moment increasingly imposes itself on consciousness as a moment in extended crisis, with one happening piling on another’ (2011: 7) – what Vigh calls ‘crisis embedded in crisis’ (2008: 13).

Crisis shapes our institutions, and how we interact with them and each other. Within the European context, anthropologists have examined a particular aspect of crisis, what has been called either the ‘retrenchment’ (e.g. Vollebergh et al 2021) or ‘reconfiguration’ (e.g. Clarke and Parsell 2022) of the welfare state. In the UK, after over a decade of austerity measures (Blyth 2013; Cohen and Bock 2017; Powers and Rakopoulos 2019) that have caused cuts in the public sector and rising poverty rates, the role of charities has expanded.

There are numerous charitable organisations supporting healthcare causes in the UK, including NHS charities. Exploring their activities offers an interesting example of how the ‘mixed economy of welfare’ (Powell 2019) works in practice, and how a historically blurry line within the NHS – between what should be state-funded, or simply an ‘add-on’ provided via charitable or voluntary means – is thought of and navigated by different people, at a time of ‘extended crisis’. Drawing from research conducted in an English city and with an NHS charity, the aim of this article is, first, to explore the role of the charity in supplementing healthcare *and* also fulfilling the role of a welfare service. While conversations and participant observation with paid staff illuminate debates on the extent to which charity and voluntarism should contribute to healthcare services, interactions with members of the public showed that volunteering with the organisation was not just about supporting the NHS, but also helped them overcome issues that might traditionally be addressed by state-funded welfare services, providing respite from various forms of precarity.

Second, the article introduces the idea of everyday pragmatism to explain people’s motivations to work, volunteer, and fundraise for the NHS. All participants, whether they were paid staff or members of the public, expressed a feeling of ambivalence about charity and voluntarism in the NHS. Many said that perhaps NHS charities should not exist – but that they were nonetheless needed, so that ‘the NHS crisis’ would not worsen. I develop this concept drawing from Davina Cooper’s work on ‘everyday utopias’ (2014) and ‘prefiguration’ (2016, 2020), and Berlant’s *Cruel Optimism* (2011), while linking to anthropological texts exploring charity and voluntarism, also in other national contexts. I aim to call attention to people’s motivations and practices to improve the present moment, while imagining, demanding and waiting for a better future.

## Is it a Crisis?

Like the term ‘neoliberalism’, the word ‘crisis’ is often bandied about in academic texts. Janet Roitman argues that ‘crisis is not self-evident’ (2014: 35) and neither is it evident when an event (that may otherwise be considered ordinary) should be read as a forewarning of crisis (2014: 49). Roitman is right to problematise the over-use of ‘crisis’ as an analytical category as it can lead to theoretical thinness, obscuring that crisis claims may have less to do with yearning for the return of a known, less difficult past and more with desiring a better, but imagined, future (2014: 9). However, I align myself with Alex Khasnabish’s (2014) reading of Roitman’s work. While recognising the importance of examining the roots and uses of the ‘crisis’ category, Khasnabish highlights that an over-emphasis on the task of defining what ‘crisis’ means can detract from material and lived conditions of injustice, inequality, and oppression (2014: 572–576). Thus, my article does not contribute to debates on what ‘crisis’ is or is not, but rather uses it as a framing concept to explore charitable and voluntarist responses to the general pressures that the UK’s welfare state is currently facing, the impacts of which are well-documented in relation to the NHS (Holmes 2023; Möller 2025: 1; Pushkar 2019: 243–244).

Khasnabish’s critique of Roitman’s *Anti-Crisis* also resonates with debates on the retrenchment vs the reconfiguration of the welfare state introduced above. Using ‘crisis’ as a framing concept allows us to examine how people navigate and resist new circumstances. Letizia Bonanno’s research with volunteers in an Athenian ‘social clinic’ (2022, 2023), within a context of prolonged austerity, provides an example of how the retrenchment vs reconfiguration debate may be reconciled. The crisis context Bonanno describes is fundamental for care practices to flourish within the solidaristic space of the clinic (2023: 1068), and the exchange and sharing of pharmaceuticals (considering drastic cuts to, and privatisation of, public healthcare) is a catalyst for new forms of solidarity to emerge. However, Bonanno is also careful not to idealise voluntarism and other grassroots movements (2022: 418), arguing that these caring communities are important but imperfect solutions to address the long-standing and multifaceted crises Greece is facing.

## NHS Charities in the Present Context

The NHS was established in 1948, as a state-funded service, free at the point of access. Before this, many hospitals were voluntary institutions that held large endowments and were funded through charitable donations (Stewart 2023). Although charitable fundraising still had a significant presence in the NHS after 1948, it was understood that this funding would not be used to finance direct healthcare provision, and instead directed to medical research and patient welfare (Bowles et al 2023). However, charity and voluntarism in the NHS went through various transformations, particularly since the 1980s, when the Conservative government eased restrictions on health authorities’ ability to raise charitable funds in the NHS (Stewart 2023: 39). Historically, there are notable disparities among NHS charities in terms of their scale and reach. Some are large organisations, such as the one this article will discuss, that raise millions of pounds in charitable income; others are small, have few resources or may fundraise for causes that are ‘less popular’, like mental health or community health (Bowles et al 2023).

At the time of writing, over 230 NHS charities exist across the UK (Stewart et al 2024: 8), most of which are associated with the umbrella organisation NHS Charities Together. Charitable fundraising by NHS charities has traditionally been carried out on a local scale, often focusing on supporting local hospitals or even specific hospital wards (Stewart 2023: 39–40). There are numerous ways in which fundraising is carried out, from bake sales to sponsored sporting events, to more creative endeavours like skydiving.^1^ Donations are spent on a range of initiatives, from improving NHS staff room facilities and distributing care packages for relatives visiting loved ones in hospital, to much more ambitious endeavours, like buying medical equipment or building infrastructure. But the financial contribution that NHS charities make to the NHS is not comparable to total government expenditure on healthcare. Charitable funding channelled to the NHS has rarely ever exceeded one percent of the government budget assigned to healthcare, and historic and regional inequalities between NHS Trusts^2^ impact the contributions that charity can make.

Nonetheless, the impact some of these organisations make (monetary and otherwise) is still significant, particularly at a time when healthcare in the UK is perceived to be in a state of emergency. Echoing Roitman’s (2014) call to treat the notion of ‘crisis’ more critically, some scholars have argued that the severity of the NHS crisis has been overstressed (Powell 2015). Indeed, there has never been a period of ‘largesse’ for the NHS. What level of funding should be granted to the health service has been a subject of debate since its inception (Greener and Powell 2024: 316), and ‘resource rationing’ within the NHS has always been commonplace. However, as mentioned, years of austerity measures and funding cuts, Brexit and COVID-19, have all severely affected the NHS. This manifests in long patient waiting lists, staff shortages, repeated industrial disputes and aging hospital facilities, which have grave implications for people’s access to healthcare, deepening already existing health inequalities. This is an important backdrop within which to situate the contemporary work of NHS charities. Further, the strength of feeling of those who volunteer, fundraise or donate to the NHS reflects the powerful affective realm that the ‘symbol’ of the NHS holds in the British imaginary (Stewart 2023), to the point that research has found that even negative healthcare experiences cannot tarnish this ideal (Stewart et al. 2026). This makes the case of charity and voluntarism in the UK’s health system particularly interesting.

## Institutional Ethnography in a ‘Post-pandemic’ World

The research on which this article is based stems from a larger Wellcome Trust funded project exploring charity and voluntarism in the NHS since 1948.^3^ I carried out research between May and October 2023, conducting ethnographic fieldwork on three visits to the English city where the NHS charity I collaborated with is based.^4^ The first two visits lasted three and two weeks respectively, while the final visit lasted one week; when not on location, I kept updated with the charity’s work via their social media, e-mail and text message exchanges with participants, and carried out online semi-structured interviews with people I met on my visits. As an anthropologist with experience of conducting institutional ethnography some years before the COVID-19 pandemic, I was met with a very different type of institution in my research on NHS charities. With the advent of remote working during the UK lockdowns of 2020 and 2021, many institutions have now acquired a ‘diffuse’ quality, no longer restricted to the confines of buildings, in-person work and defined temporal routines. The ‘deep hanging out’ that anthropologists are so reliant on can be challenging in these new circumstances, and I found myself adapting my approach similarly to what Gökçe Günel and colleagues have termed ‘patchwork ethnography’ (Günel et al 2020; Günel and Watanabe 2024), where year-long fieldwork is replaced by short-term, rigorous visits and complemented by other methods (such as online ethnography).

I negotiated access in stages, first via online calls with charity staff, and then again in person. Video calls and e-mail introductions before my first visit in June 2023 offered valuable glimpses into the daily work of paid staff and volunteers, but it was far from a full picture of the institutional setting I would encounter in person. Differently to my past experiences, I did not find myself getting to know participants within a busy office environment (the charity’s headquarters were predominantly vacant, used as a storage facility or an occasional quiet space for online meetings), or while embedding myself in predictable routines.

In the first days after my arrival in June 2023, I struggled to meet anyone in person due to paid staff’s workload demands, which were typically faced from their home offices, and I could not speak to volunteers before being formally introduced by a member of staff. I spent those days on a self-guided tour of locations where traces of the charity’s work were easily observed; hospital hallways displayed artworks acquired through donations, posters thanked supporters for specific fundraising achievements, upcoming campaigns were advertised on billboards by main roads. After this slow start, I finally became immersed in the main event I was invited to be involved in – a three-month-long fundraising campaign across the city, part of an ambitious capital appeal to build new hospital infrastructure. It was at around three in the morning on the night I helped set up for the launch event of this endeavour, alongside staff members and volunteers, that I realised understanding this organisation and those affiliated to it was about almost always being in motion. Conversations happened while setting up for events, while unloading boxes of supplies for NHS staff care packages from the back of trucks, in between deciding how to price items in charity shops, or while manning the hot drinks stall at a fundraising event outside a hospital. The variety of contexts I was invited into, as well as the diverse demographic of the city, meant that I met people from a wide variety of backgrounds, both paid staff and volunteers. Thirteen interviews were carried out in person, while eleven were conducted online, although in some cases conversations would start in person and carry on in a videocall, or conversely begin online and develop when I was in the field. Paid staff I spoke to and volunteered with were young (mid-20s to mid-30s) or mid-career professionals (40 and above), and were all white British, with one exception. The volunteer population was more varied, as will be explained below; the majority were retirees or university students (both British and international students). Online interviews were chosen by participants for practical reasons, mainly because it was easier for them to have longer conversations from their own homes; the normalisation of videocalls during the COVID-19 pandemic has also meant that online conversations, while not replacing in-person interactions, can still help researchers develop closeness and rapport with participants (Keen et al 2022).

While conducting this research, I had to reflect at length about my visceral reactions to what is considered a healthcare ‘extra’ or ‘necessity’, both in general discourse as well as in participants’ accounts, at times finding myself slightly disconcerted by some of the things that rely on the charity to be funded. Philanthropy and charity are generally contested spheres, with critics arguing that they serve to uphold the status quo (e.g. Littler 2018: 131–133) and impede solidarity (e.g. Spade 2020). These are perspectives I tend to gravitate towards, and which anthropology generally adopts, also in relation to the field of humanitarianism; as Peter Redfield and Erica Bornstein contend, ‘anthropologists are often of two minds when it comes to “doing good” and more comfortable with the stance of critique rather than endorsement’ (2010: 21). This instinct is, in part, informed by the sense that within the context of ‘extended crisis’ of the UK’s welfare state generally, and the NHS particularly, accepting charity and voluntarism as a solution to address shortcomings in service provision is letting the state ‘off the hook’. The sustainability of this solution (like for humanitarianism) is also questionable, particularly if people have less money to donate or less time to volunteer, as some organisations have experienced during the UK’s ‘cost of living crisis’.

However, especially during field visits, I also found myself immersed and often persuaded by the mission of NHS charities, and the passion with which people engaged with this organisation in various capacities. Like in Bonanno’s work cited above, the structures of care and solidarity that emerged in my fieldsite are a result of the complex relationship between the desire to ‘alleviate suffering and a social organisation designed to improve collective conditions of life’, as Craig Calhoun has argued for the case of humanitarianism (2008: 90). These two aims, at times, seem to contrast each other, yet they are also inevitably complementary. Understanding, furthermore, that charity and voluntarism in the NHS have a long history (Stewart et al 2024), separate from the context I have described, reveals that ‘supporting the NHS’ means more to people than merely responding to crisis.

## Charity: Just ‘the Cream on Top’?

The NHS charity this article discusses is an ambitious and growing organisation, with a team of about 50 paid staff, a rota of over 400 volunteers and a total gross income of almost £8 million in the year I conducted fieldwork. Their aims range from supporting local NHS staff and patients, helping to improve local health outcomes, to becoming leaders in supporting specific areas of healthcare provision. Thus, their fundraising strategy spans a varied and wide range of activities and initiatives, like sporting events, fairs, balls and charity shops.

It was a busier day than usual for the charity the day I met Tom^5^ in summer 2023, as their new, big fundraising appeal was about to launch. After meeting me for a quick coffee break, Tom rushed off to be interviewed by the BBC in his capacity as Head of Fundraising at the charity, to discuss this new campaign. It had almost been a week since I had arrived in the city, one of the largest in England, and it was exciting to finally see, in person, the number of initiatives that the charity was involved in. Are charitable and voluntary intervention simply ‘adding value’ to existing healthcare services, or playing an essential role in funding what the government cannot, or will not? I asked Tom his thoughts. He said:

I once heard, it was the Chair of [another NHS charity], and he said, ‘You know, think of the NHS and charities like a bottle of old milk. You know, milk as in you have the cream on the top, and the charity’s the cream on the top.’ He said, ‘But the problem is we were donated arts, pieces of artwork for the wards and,’ he said, ‘the walls were looking good, but there were no blankets on the beds.’ So it’s a question about priorities as well. But I think the line’s getting a bit more blurred between what charities will fund and what they used to fund.

NHS charities are historically known for providing what Tom described as ‘the cream on top’, the ‘extras’ or ‘comforts’ that add to patients’ or NHS staff’s experiences (Stewart and Cresswell 2023). However, at present, NHS charities seem to face increasingly difficult decisions about how to allocate funding, as Tom went on to explain:

I think in previous [NHS] Trusts I’ve worked in there was a lot of pressure on the charities to provide, what I consider to be, capital budget […] Yeah, it’s a challenge and it’s symptomatic of the pressures on the NHS. It’s well documented what those pressures are. I think […] it’s only going to get worse unless there’s significant change in the funding structure of the system. We want more doctors, we want more nurses; that all costs, so where are those funds going to come from? It can’t be charitable.

Other staff expressed this preoccupation by defining the boundaries of their work, both being careful not to overstate the role of the charity in adding essentials to the local NHS but also acknowledging their contributions were no longer limited to ‘putting art on walls’. It is in these discussions that I perceived some of the ambivalence introduced earlier. Grace, another staff member, said:

I think whenever you work in the charity sector, whatever charity it is that you’re working for, the ultimate goal would be that the charity didn’t exist […] but it is scary to think how the [NHS] staff would struggle to deliver the care that they give, and what our experiences would be as patients coming into the hospital if things weren’t funded [by our charity].

In the months to come, I would hear a version of this viewpoint from many participants, both paid staff and members of the public: that NHS charities should perhaps not exist, but that if they did not, the consequences would be seriously felt by NHS patients and staff. Similarly to those who worked for the charity, volunteers’ ambivalence seemed to be mixed with a sense of urgency, which was related to me as one of the key motivators to support the organisation. In the words of Laura, a charity shop volunteer:

I don’t think that we should be necessary, however, I think it’s absolutely brilliant that we’re here because it does help, and especially things, like new equipment that you can’t … you can’t expect the NHS to be trialling new equipment on the money that they’ve got, it’s just not feasible. So things like that I think are really good […] and things like helping staff, I think that should be done on the NHS, but I’m happy that they get the support from us.

Participants’ accounts point to an urgency that seems prolonged, and where responding urgently to a situation becomes more embedded in daily practice, rather than being an exceptional action (Bandak 2023: 6). It is in these circumstances, I argue, that everyday pragmatism manifests. Participants’ narratives and practices show a motivation to act in the present intertwined with imagining, or demanding, a better future. As Maggie, who volunteered with the charity in different capacities, told me:

I’m very cross that this government, particularly, is underfunding, to my view, the NHS […] So part of me is cross that charities have to have a role in funding the NHS, but on the other hand I feel it’s my responsibility, really, to contribute wherever I can […] And I’ve got the time and the ability to make a contribution, so I will do that, and try and make my political voice heard at the next election.^6^

While exploring participants’ motivations as everyday pragmatism, which I will elaborate on below, I was also struck by a second role NHS charities seem to fulfil, as a sort of ‘support service’ for volunteers. This added benefit is a common feature of charitable and voluntary activity, as also explored in work carried out in other contexts, like Liisa Malkki’s research with Red Cross ‘volunteer friends’ in Finland (2015), Rosie Read’s work with hospital volunteers in the Czech Republic (2010, 2014) and Andrea Muehlebach’s ethnographic account of socialist and Catholic volunteers in Italy (2012). The community of elderly knitters that Malkki encounters in her ethnography, for example, take part in the ‘worldwide helping’ (2015: 151) of the Red Cross – their craftworks (hand-knitted ‘Mother Teresa blankets’ to donate to ‘the needy’ via humanitarian organisations) are made ‘in aid of others’ (2015: 157). However, Malkki notes also that ‘helping helps’, in that it ‘can enable forms of sociality and conviviality that may, in turn, offer some degree of protection against loneliness, social isolation, and asocial time’ (2015: 134). There is, further, dignity in giving, and in making things with one’s hands (2015: 161). Importantly to this article’s discussion, Malkki describes some of the loneliness and isolation that her participants experience as a ‘crisis of ordinariness’, no longer exceptional but rather embedded in their everyday lives (2015: 139, 164). In Read’s ethnography, the state and voluntary sector are engaged in a ‘productive cooperation’ (2014: 94) rather than an antagonistic relationship. Yet, while hospital volunteering predominantly provides patient support and helps address issues of scarcity, Read finds that it is also ‘an opportunity for self-realisation and personal fulfilment’ (2010: 560–561). In Muehlebach’s work, volunteers’ ‘relational work’ is enacted as a dual act of care: care for the self is achieved through caring for others (2012: 8). However, this voluntarism is also rooted in crisis, being ‘part of a much larger resignification of the meaning of work in a Europe confronting the spectre of growing unemployment rates and the growth of an increasingly precarious, low-wage labour market’ (2012: 8). For some of the people I met, ‘helping the NHS’ in its time of crisis was a motivating factor to volunteer, yet other crises also emerged in their accounts as reasons to fulfil these roles.

I first became aware of volunteers’ varied backgrounds by talking to paid staff. I met Gemma, the manager of the organisation’s charity shops, on my first fieldwork visit. She advised me to come at a time when the store would be quieter, so we could have a chance to speak properly. We met on a weekday morning; she gave me a tour of the charity’s flagship shop while introducing me to some of the volunteers who were there. Sitting in her office, she talked animatedly about her role, and particularly her transition from working as a manager in corporate retail to a similar position within the charity. Gemma explained that, as well as the volunteers who are, in her words, the ‘usual suspects’ (typically white, middle-class retirees), there was also a ‘new demographic of volunteers’ – people with additional support needs, who are unable to access paid work experience for various reasons, or who want to improve their confidence or English language skills (like the many international students living in the city, for example). She told me:

I’ve never worked with volunteers before […] I’ve always worked with paid retail staff. So the way in which you speak to volunteers, you manage volunteers, the way you are with volunteers is completely different. I’ve also found that I’ve become a bit of a social worker in a way. You have a lot of volunteers that have needs that are more special to them or you have a lot of volunteers that need that extra bit of care and attention […] But I’m a people person so I have empathy […] So yeah I love it, I love working with the volunteers and I just think they’re amazing.

Her comment that in this role she had to become ‘a bit of a social worker’ surprised me. To better understand this, I volunteered alongside and got to know some of the people in this ‘new demographic of volunteers’ Gemma described, many of whom told me some of the challenges that volunteering had helped them overcome. Mental health emerged as a particularly prominent topic, and, on occasion, volunteering was related to me almost as a matter of life and death. This was the case for Tessa and Robert, for example. Tessa, who had volunteered in one of the charity shops for almost two years, recounted a traumatic experience that had impacted her ability to work, and volunteering as part of her recovery. Explaining that becoming part of the charity shop team helped her overcome feelings of isolation and loneliness, she told me, ‘it [volunteering] has just brought a new me back’. Robert shared a similar story about overcoming a period of bad mental health, and his motivation to ‘give back to the NHS’ – showing gratitude for care received during hospital stays – as a reason to volunteer at one of the charity shops. Similarly to Tessa, he described his time as a volunteer as ‘lifesaving’, and recounted the benefits that being part of this charity shop team had brought to his weekly routine.

Gemma’s comment about being ‘a bit of a social worker’ thus became clearer the more I spoke to volunteers. Other paid staff at the charity shared Gemma’s view. Heather, for example, explained that she found supporting people to be one of the most rewarding aspects of her job as a volunteer coordinator:

We find so many volunteers come to us after they’ve been marginalised or made to feel like they’re not worthy of paid employment, or being part of a community. For me a massive thing is guiding them through that journey, picking them up when they’re sometimes at their lowest, and making them feel like they do belong and they have a role in society […] It opens so many doors to so many people, and it’s a really lovely thing to see.

In exploring the ‘dual’ role that this NHS charity plays in supporting healthcare, and also being a welfare service of sorts to those who volunteer for it, one might argue that organisations like these are not simply providing ‘the cream on top’; this can be said not just of NHS charities, but of the UK charity sector generally, where the last decade, particularly, has seen an expansion of charitable intervention in myriad areas, from social care and housing to emergency food provision. Some point to this pattern as a retraction of the welfare state, evidence that ‘the consequences of austerity have gone beyond short- or even medium-term material impact. They have revealed the long-term incapacity of the state to confront urgent problems’ (Cohen and Bock 2017: 3). Others suggest that the expansion of charity and voluntarism ‘is more than a mere crutch for welfare state retrenchment … it is also mobilized for its own sake’, because it endorses ‘a mode of sociality that has long been at the heart of liberal conceptions of how society is, and should be, organized’ (Clarke and Parsell 2022: 308). Insa Koch and Deborah James similarly contend that this reconfiguration of the welfare state has ‘produced new forms of ethical relationships’ (2022: 1), which is Muehlebach’s core argument in *The Moral Neoliberal* (2012). We may view charity and voluntarism thus not only as responses to diminished state involvement, what Nikolas Rose termed ‘governing through community’ (1996), but also as spheres of activity where hope can be generated (Cloke et al 2017: 705) and ‘extended crisis’ can be made sense of.

## Temporality, Imagination and Everyday Pragmatism

Kate and I rested at the charity’s headquarters, after a morning spent alongside other staff unloading boxes from a delivery truck on to metal crates, which we then transported to a storage room in one of the hospitals. These boxes contained juice boxes, iced coffee drinks, chocolate milk – all donations intended to fill care packages for NHS staff, together with other items. It was rare to have a conversation with Kate while sitting down; in the time I had spent with her, it was common to be on the go, at different hospital sites while she coordinated fundraising activities, or helping set up for events around the city. Like in most of the charity sector, work at this organisation is fast-paced and reactive, yet many of their goals will take a long time to be realised. Kate reflected on this, for example, in relation to one of their long-term projects, a capital appeal to build new infrastructure:

The capital appeal is launching […] It’ll take forever to build like these things do, but the fact that it is coming is really exciting. But one thing on that is that we were, for a while, things were being put on hold because, ‘Oh well, we’re getting a new [building]…’ ‘When we get a new [building]…’ But we’ve now come to the understanding that actually it’s going ahead. It’s going to be open in, what? Ten years, something like that, seven years, 2030 I think they’re saying […] That’s still seven years of [patients] coming through these current doors. So we still need to be making their experience as good as possible.

Kate’s comments capture the sentiment of what I describe as everyday pragmatism. It is a motivation to act in the present moment where urgency has become ‘ordinary action’ (Bandak 2023), where practices that might seem antithetical to animating structural change are implicated in imagining a better future, and ‘new forms of citizenship and social solidarity’ (Clarke and Parsell 2022: 311) can emerge.

I draw on Cooper’s work on ‘everyday utopias’ (2014) and ‘prefiguration’ (2016, 2020) to think of everyday pragmatism. In *Everyday Utopias*, Cooper describes the ‘everyday’ as defined by ‘a pragmatism oriented to survival and to doing the best one can; of establishing, promoting, and maintaining internal rules, systems, adjudicative structures, and etiquette conventions’, and points to an ‘ethos of … getting things done’ (2017: 7). Prefiguration, similarly, ‘involves enacting desired economic, social, and political changes rather than awaiting their “right time”‘ (2016: 328). Kate’s comment that patients’ experiences need to be ‘as good as possible’, presently, and in the next years before the new infrastructure is built, exemplifies the ‘prefiguration’ Cooper describes. Indeed, Kate laments that different initiatives have been put on hold because ‘the right time’ – the inauguration of this new infrastructure – has not yet arrived. The question of the scale of charitable intervention did not seem to matter much in these considerations, however; acting in the here and now could be something ‘small’, like making care packages for NHS staff, or something much more ambitious, like building new infrastructure. It is not about scale, but about *doing*. In the words of Laura, the charity shop volunteer introduced earlier:

The things that [the charity] do tend to be things that the NHS just can’t afford, like perhaps trialing new equipment. [They] also help staff to have […] an easier time of doing long shifts and things like that, so we have things like the occasional parcels for them […] from my perspective I just think that the NHS itself is probably struggling more than a lot of things, in terms of the country and where the money is going. So I just think that anything that we can do to help, it’s got to be good.

Thus, returning to the question of whether NHS charities provide healthcare ‘extras’ or ‘necessities’, the answer for many of the people I met seemed to be that NHS charities currently do both. It also seemed that determining this does not ultimately matter – any help the NHS gets has ‘got to be good’. The collective efforts of paid staff and volunteers (from volunteering in charity shops, handwriting thank you cards to donors, to running successful large-scale fundraising campaigns year-round) all contributed to improving present NHS staff and patient experience; funds raised might be used to buy comfortable chairs for chemotherapy patients, paying some of the hospital staff’s salaries, or funding medical research.

The sustainability of the charity’s expanding role was an underlying concern, and inherent to the ambivalence expressed by my participants. However, this is not unique to this case. Bonanno showed that some acts of care in the social clinic in Athens were also riddled with contradictions. One of her participants shared that she did not believe in the biomedical establishment, favouring instead alternative therapies, but still thought it was important to ensure people in the community had access to pharmaceuticals (2023: 1065). Others, when questioned about the safety of informally sharing medicines with people, said: ‘We know it can be risky and dangerous! Distributing meds is all we can do for now, *it is a temporary fix*… […] *We’ll stop giving the meds when the crisis is over*’ (2022: 422, emphasis added). Like Bonanno’s interlocutors, those I met over the course of my fieldwork emphasised the need to (imperfectly) act in the present, while voicing a vision for a better future.

However, unlike in Cooper’s *Everyday Utopias*,^7^ it would be difficult to argue that NHS charities create spaces where radically different alternatives to the status quo are generated. Rather, as in Cooper’s later work on ‘transformative state publics’, people who work, volunteer and fundraise for NHS charities are ‘publics that take a less obedient shape’ (Cooper 2016: 321; see also Stewart 2021) but whose practices might still align with established norms and systems.^8^ Paid staff, who work in a highly professionalised sector, needed to balance their feelings of ambivalence with the charity’s strategic goals to grow as an organisation. For volunteers, overcoming personal crisis was just as important as supporting the NHS and, further, love and gratitude for the NHS often featured as participants’ key motivation to volunteer. And, while both paid staff and volunteers constantly reflected about the pressures the NHS is facing, and their desire to make things better presently and in the future, the possibility that engaging in charitable work might be contributing to the retrenchment of the welfare state did not emerge in their accounts.

This seemingly paradoxical situation – engaging in practices that appear to not align with hoping for and/or demanding alternatives – is key in discussions where the ‘reconfiguration’ (rather than just the ‘retraction’) of the welfare state is examined. It also contextualises the ambivalence I have mentioned throughout this article, and that is evident in other ethnographic accounts of charity and voluntarism in Europe. Muehlebach, for example, describes the mobilisation of morality in neoliberal Italy as ‘the very vehicle through which subjects … get drawn into processes they might not be in agreement with’ (2012: 5), yet that also ‘allow[s] those uneasy or explicitly critical of neoliberalization to render … novel practices of citizenship meaningful and graspable in their own terms’ (2012: 5). In the UK context, Paul Cloke and colleagues describe food banks as ‘ambivalent spaces of care’, where voluntarism ‘should not be read as an uncritical endorsement [of charity]’ but as sites that ‘will usually represent a complex amalgam of ambiguous and contradictory dynamics involving charity, solidarity and civic spirit’ (2017: 715). They also examine people’s motivations to act urgently in a time of austerity-driven crisis, arguing that food banks ‘can serve as potentially virtuous arenas of common life’, in which voluntaristic responses ‘can lead eventually to political and ethical ruptures’ of the status quo (2017: 720).

The contradictions described in my ethnography and the texts above can also be understood following Berlant’s definition of cruel optimism as ‘the condition of maintaining an attachment to a significantly problematic object’ (2011: 24). An initial reading of Berlant’s work may suggest a defeatist outlook, yet their work is also preoccupied with illuminating how people make sense of, and ‘organise’, a present that is defined by prolonged crisis. Cruel optimism, they contend, is also ‘a scene of negotiated sustenance that makes life bearable as it presents itself ambivalently, unevenly, incoherently’ (2011: 14). Charity seems to have acquired a ‘cruel optimist’ quality, as different crises expand, interlink, and become further entrenched. They are imperfect solutions that genuinely seek to redress inequalities, but are deeply constrained by structural issues. When the temporality of crisis becomes a settled ‘social state’ (Vigh 2008), responses to it become part of daily practice. Everyday pragmatism thus describes a motivation to act not only despite the circumstances, but in acknowledgement of them.

## Conclusion

This article opened with questions about the expanded role of charity and voluntarism in a time of ‘extended crisis’ in the UK, linking to broader debates on whether this change signals a ‘retrenchment’ or ‘reconfiguration’ of the welfare state. Taking the example of a large English NHS charity, I have shown that NHS charities can no longer be considered to simply be ‘the cream on top’ but rather are increasingly contributing to providing healthcare necessities. I argue that this demonstrates charitable organisations’ growing significance in the UK by also highlighting the ‘dual role’ that the NHS charity plays, of supporting the NHS while also being a ‘safety net’ to some of the volunteers who engage with it.

Further, I have discussed participants’ ambivalence with regards to the role of charity and voluntarism in the NHS who, despite thinking that NHS charities should perhaps not exist, believed they needed to act in the present to alleviate the current crisis, while imagining, demanding and waiting for a better future. I have developed the notion of everyday pragmatism to describe this motivation to act in the present despite, and in acknowledgement of, various structural constraints and contradictions. While constantly navigating the question of what should or should not be charitably funded in the NHS, paid staff not only sought to improve present NHS patients’ and staff’s experiences while hoping for better times but also created a supportive community for volunteers. Some volunteers, motivated by the aim of supporting, or ‘thanking’, the NHS, also found that volunteering provided them a space of cooperation and safety. Read like this, charitable intervention in the NHS might be understood, in Berlant’s words, as ‘a stubborn refusal to give out’ so that ‘optimism might not be cruel at all, but the bare minimum evidence of not having given up on social change’ (2011: 259).

## Notes

1 For an account of the many fundraising activities supporting the NHS during the early days of the COVID-19 pandemic, see Stewart (2023: 43–45) or Stewart and colleagues (2024).

2 NHS Foundation Trusts were established during the New Labour years (1997–2008), as entities independent from the central Government, aiming to promote local autonomy and increase patient involvement (Gorsky 2008: 447–448). There are more hospitals than NHS Trusts in the UK, as one NHS Trust may support more than one hospital within a specific region.

3 Grant number 219901/D/19/Z.

4 Ethical approval to conduct this research was obtained by the project’s principal investigator in May 2022, through the University of Strathclyde’s School of Social Work & Social Policy’s Ethics Committee.

5 All participant names are pseudonyms.

6 This interview was carried out during Rishi Sunak’s tenure as UK Prime Minister between 2022 and 2024 (Conservative administration). The Labour Party’s general election victory would come about a year after this fieldwork encounter, in July 2024.

7 Davina Cooper explores practices in six ‘everyday utopian sites’ (2014: 2): a Local Exchange Trading Scheme (LETS) in England, a lesbian bathhouse in Toronto, Speaker’s Corner in London’s Hyde Park, public nudist communities, equality governance in an English local council, and Summerhill School (a school/home where students are encouraged to ‘self-regulate’).

8 Promoting voluntarism, for example, was key in policy rhetoric that elevated ‘civic responsibility’ during Conservative Prime Minister David Cameron’s time in office (2010–2016), captured in the vision of the ‘Big Society’ where communities, rather than the government, would be ‘the first port of call in meeting social needs’ (Mohan 2013: 5).
